# Crosstalk between the tumor immune microenvironment and metabolic reprogramming in pancreatic cancer: new frontiers in immunotherapy

**DOI:** 10.3389/fimmu.2025.1564603

**Published:** 2025-04-28

**Authors:** Taijin Shi, Xiaoyan Cui, Junlin Wang, Guangqia Liu, Jiayin Meng, Yingjie Zhang

**Affiliations:** ^1^ College of Medicine, Shandong University of Traditional Chinese Medicine, Jinan, China; ^2^ Pharmacy Department, Jinan Huaiyin People’s Hospital, Jinan, China; ^3^ Department of Pharmacy, Shandong Second Provincial General Hospital, Jinan, China; ^4^ Department of Pharmacy, Jinan Licheng District Liubu Town Health Centre, Jinan, China; ^5^ Department of Pharmacy, Jinan Second People’s Hospital, Jinan, China

**Keywords:** PC, time, metabolic reprogramming, mechanisms, immunotherapy

## Abstract

In recent years, the incidence and mortality of pancreatic cancer (PC) are increasing year by year. The highly heterogeneous nature of PC, its strong immune escape ability and easy metastasis make it the most lethal malignant tumor in the world. With the rapid development of sequencing technology, the complex components in the tumor microenvironment (TME) of PC have been gradually revealed. Interactions between pancreatic stellate cells, tumor-associated fibroblasts, various types of immune cells, and cancer cells collectively promote metabolic reprogramming of all types of cells. This metabolic reprogramming further enhances the immune escape mechanism of tumor cells and ultimately induces tumor cells to become severely resistant to chemotherapy and immunotherapy. On the one hand, PC cells achieve re and rational utilization of glucose, amino acids and lipids through metabolic reprogramming, which in turn accomplishes biosynthesis and energy metabolism requirements. Under such conditions, tumorigenesis, proliferation and metastasis are ultimately promoted. On the other hand, various types of immune cells in the tumor immune microenvironment (TIME) also undergo metabolic reprogramming, which leads to tumor progression and suppression of anti-immune responses by inhibiting the function of normal anti-tumor immune cells and enhancing the function of immunosuppressive cells. The aim of this review is to explore the interaction between the immune microenvironment and metabolic reprogramming in PC. The focus is to summarize the specific mechanisms of action of metabolic reprogramming of PC cells and metabolic reprogramming of immune cells. In addition, this review will summarize the mechanisms of immunotherapy resistance in PC cells. In the future, targeting specific mechanisms of metabolic reprogramming will provide a solid theoretical basis for the development of combination therapies for PC.

## 1 Introduction

PC is the most malignant tumor, and the 5-year survival rate of patients is almost only about 10% (1). In the past 20 years or so, the incidence of PC has risen almost threefold, and the number of patients is even more than 400,000 cases (2). As the third most common cause of cancer-related deaths, PC is projected to rise to second place in its lethality in the next decade. The study found. Early-stage PC usually lacks obvious clinical symptoms, and patients only show non-specific symptoms such as fatigue, weight loss and abdominal pain in the early stage (3, 4). Therefore, once PC is diagnosed, the vast majority of patients have already entered the advanced stage. Meanwhile, PC is highly heterogeneous, easy to metastasize and highly resistant to various treatments, which makes its mortality rate remain high (5). The latest epidemiological findings: half of the PC patients have metastasis at the time of diagnosis, among which liver metastasis is the most common. This makes treatment even more difficult. Patients with advanced metastatic PC usually survive for less than a year, and those who do not receive treatment survive for only six months (6, 7). It is therefore crucial to analyze the mechanisms of PC development and to find markers for early diagnosis.

TIME is a complex microenvironment formed by the interaction of tumor cells with immune cells, stromal cells and so on. It plays an important role in the process of tumorigenesis, proliferation and metastasis (8). In TIME anti-tumor immune cells such as T cells, NK cells, etc. can recognize and attack tumor cells under normal conditions and play anti-tumor roles. And due to the effects of metabolic reprogramming, epigenetic modification, etc., the normal function of anti-tumor immune cells will be significantly inhibited. Tumor cells, on the other hand, induce the proliferation and infiltration of immunosuppressive cells (e.g., Tregs, MDSCs, M2-type macrophages), highly express immune checkpoint molecules such as PD-L1 and CTLA-4, and ultimately form an immunosuppressive microenvironment (9). It can help tumor cells evade immune surveillance and promote tumor cell growth, proliferation and drug resistance. Metabolic reprogramming is a key feature of PC. Since the discovery of the Warburg effect, scientific researchers have identified multiple metabolic reprogramming in tumor cells. Cancer cells, even when well oxygenated, they still rely on glycolysis to produce ATP, which in turn is used to synthesize various biomolecules (10). With the rapid development of sequencing technology, the interplay networks of tumor metabolism are gradually revealed. They play a key role in promoting tumorigenesis and development (11). Further research has revealed that, regardless of the type of cancer cell, they usually consume glucose by means of glycolysis rather than by means of oxidative phosphorylation. This unique approach produces a massive buildup of lactic acid. The massive buildup of lactic acid, in turn, further promotes immune escape of tumor cells through lactylation and other means (12, 13). Therefore, metabolic reprogramming is crucial for the processes of tumor cell growth, proliferation and immune escape. Furthermore, metabolic reprogramming is not only found in tumor cells, but they are also able to regulate the biogenesis and energy acquisition of immune cells, which ultimately promotes the progression of tumorigenesis and suppresses anti-tumor immune responses (14, 15). These cells that have undergone reprogramming can become severely resistant to various therapeutic agents, resulting in severely poor patient survival and quality of life (16).

Although there are similar reviews published on the crosstalk between metabolic reprogramming and immune microenvironment. This paper is intended to focus on the interaction between the immune microenvironment and metabolic reprogramming in PC. The paper will first summarize the metabolic reprogramming of PC cells, along with a comprehensive summary of metabolic reprogramming in immune cells. In addition, this paper will also delve into the clinical efficacy of the combination of metabolic reprogramming and immunotherapy. In the future, by deeply revealing the metabolic and immune crosstalk mechanisms between PC cells and tumor microenvironment (TME), it will not only help researchers to reveal the mechanism of action of PC progression, but also provide new ideas for developing new diagnostic and therapeutic approaches.

## 2 Metabolic reprogramming of PC cells

### 2.1 Glucose metabolism in PC cells

PC cells enhance glucose metabolism through extremely complex and fine-grained metabolic reprogramming, which in turn promotes PC cell proliferation and metastasis. Under aerobic conditions, PC cells actively undergo aerobic glycolysis: the Warburg effect. It is as long as manifested by the high expression and translocation of glucose transporter protein 1 (GLUT1) on the cell membrane, along with a substantial increase in glucose uptake by tumor cells. During this process, the expression of hexokinase 1/2 (HK1/2) and lactate dehydrogenase A (LDHA), which promote glycolysis and its conversion to lactate, are significantly upregulated (17). Lactate substantially inhibits normal anti-tumor immune responses and enhances metastatic and drug-resistant properties of tumors. Furthermore, Pyruvate dehydrogenase kinase 1(PDHK1) in PC cells inhibited the formation of the pyruvate dehydrogenase (PDH) complex, which in turn inhibited the oxidative phosphorylation (OXPHOS) process in mitochondria. Through the above pathways, the Warburg effect was eventually significantly enhanced (18). The non-oxidative pentose phosphate pathway (PPP) and HBP are significantly upregulated in pancreatic ductal adenocarcinoma (PDAC) cells (19, 20). PPP feeds anabolism such as DNA synthesis by upregulating ribulose-5-phosphate-3-epimerase (RPE) and RPIA. HBP feeds the synthesis of proteins and substrates for lipid glycosylation by upregulating glutamine and fructose-6-phosphate amidotransferase-1 (GFPT1) (21, 22). HBP is also upregulated by upregulation of GFPT1. HBP is also upregulated by upregulating the amino acid pathway (AAP). Both ultimately promote tumor cell progression and drug resistance. Meanwhile, overexpression of nicotinamide phosphoribosyltransferase (Nampt) in PC cells utilizes nicotinamide adenine dinucleotide (NAD), which in turn maintains high levels of glycolytic processes in the cell. These metabolic reprogramming processes are primarily driven by mutations in key genes. Mutant KRAS promotes the expression of glucose transporter type 1(GLUT1) and key glycolytic enzymes by activating downstream effectors (PI3K and RAF). It also drives phosphoglycerate kinase 1(PGK1) to inhibit OXPHOS. KRAS mutations also play a key role in metabolic reprogramming. However, some glycolysis-related enzymes are still highly expressed after KRAS inactivation, suggesting that some of these processes can be carried out independently of KRAS (23).TP53 mutations significantly enhance the Warburg effect by increasing the expression of paraoxonase-2 and inhibiting TP53-induced glycolysis and apoptosis regulator (TIGAR) (24). They also maintain a certain level of glycolysis by stabilizing glyceraldehyde-3-phosphate dehydrogenase (GAPDH) in the cytoplasm. In addition, hypoxia-inducible factor-1 (HIF-1) upregulates the expression of glycolysis and the HBP-related enzyme GFPT2 under hypoxic conditions and inhibits PDH, impairing mitochondrial oxidation. Muscle isomerase type 2 pyruvate kinase (PKM2) expression (25, 26). On the other hand, varies according to nutritional conditions and is inhibited in low-glucose environments, which promotes autophagy and biomolecule accumulation, aids in tumor cell proliferation and reduces oxidative stress. Transcription factors such as Forkhead box protein M1 (FOXM1) and deacetylation modification of LDHA are also able to regulate the glycolytic process (27). Tumor cells produce lactate in large quantities through the Warburg effect, which not only provides energy for tumor cells, but also supports a series of metabolic reactions by transferring lactate between cells through the “lactate shuttle”. In addition, lactic acid-induced lactation modification affects gene expression and function by regulating the modification of histones and non-histone proteins, and ultimately promotes metabolic reprogramming and immune escape from tumors (28, 29). Chen and colleagues et al. found that nucleolus and spindle-associated protein 1 (NUSAP1) promotes glycolytic metabolism by binding to c-Myc and HIF-1α to form a transcriptional regulatory complex located in the promoter region of lactate dehydrogenase A (LDHA). In addition, it was found that lactate produced from glycolysis was able to inhibit NUSAP1 protein degradation by way of lysine lactylation modification, which in turn upregulated NUSAP1 expression. Such unique regulation induced the formation of NUSAP1-LDHA-glycolysis-lactate feedback pathway, which ultimately promoted the transfer and metabolic reprogramming of PDAC (30). In PDAC, there exists a significant elevation of histone lactylation levels in tumor cells, especially H3K18 lactylation (H3K18la). Li et al.’s study found that enhanced glycolysis in PDAC cells leads to a large accumulation of lactate, which in turn promotes an increase in the level of H3K18la, as well as activating the transcription of the cell cycle-related genes TTK and BUB1B. These two genes not only promoted tumor cell proliferation, but also further enhanced glycolysis and lactate levels by upregulating P300 and activating lactate dehydrogenase A (LDHA), ultimately forming a positive feedback loop (31).

PC cells significantly enhance the efficiency of glucose metabolism in tumor cells through the aforementioned complex and fine-grained metabolic reprogramming pathways and sophisticated regulatory networks. Together, these mechanisms promote the survival, proliferation and progression of PC cells.

### 2.2 Amino acid metabolism in PC cells

In PC cells, amino acid metabolism likewise undergoes a high degree of reprogramming to promote rapid tumor proliferation and progression. Among them, amino acid metabolism mainly involves tryptophan metabolism, glutamine (Gln) metabolism. In PDAC, tumor cells are made more inclined to metabolize Gln by enhancing the activity of aminotransferases such as GOT1 and GOT2, which in turn, through activation of the Kras pathway. The above processes ultimately promote the growth and proliferation of PDAC cells (32). In PC cells, tryptophan metabolism occurs mainly through the kynurenine (Kyn) pathway (33). About 95% of tryptophan is catalyzed by tryptophan-2,3-dioxygenase (TDO) and IDO to generate Kyn and other biologically active metabolites and Kyn. In turn, they regulate the tumor cell cycle, promotes antioxidant responses, and influences gene expression through aromatic hydrocarbon receptor (AHR)-mediated signaling, which ultimately promotes the survival and proliferation of tumor cells (34). In addition, PDAC cells enhance tryptophan degradation by upregulating the expression of IDO and TDO to further meet the metabolic demands of tumor cells. In addition, glutamine (Gln) metabolism plays a critical role in PDAC (35). Driven by KRAS mutations, Gln is efficiently utilized through a non-classical metabolic pathway. Gln is first deaminated by glutamine synthetase 1 (GLS1) to generate glutamate (Glu), which is then catalyzed by glutamate aminotransferase 1 (GOT1) in the cytoplasm and reacts with oxaloacetic acid (OAA) to generate aspartic acid (Asp) and α-ketoglutarate (α-KG) (36, 37). The generated OAA is converted to MDH1 and then oxidized by ME1 to generate pyruvate and NADPH, which maintains the intracellular reducing state and protects against reactive oxygen species (ROS) accumulation. In addition, the KRAS-driven non-classical Gln metabolic pathway proceeds through arginine methylase 1 (CARM1)-mediated MDH1 methylation, which is inhibited to regulate oxidative stress and ultimately to meet the changing metabolic demands of tumor cells (38). Upon long-term inhibition of GLS1, PDAC cells restored the production of Gln-derived metabolic intermediates by up-regulating glutamine synthetase 2 (GLS2), demonstrating metabolic pathway adaptation (39). In addition to tryptophan and glutamine, PDAC cells support tumor growth and survival by reprogramming other amino acid metabolic pathways. In proline metabolism, PDAC cells overexpress proline oxidase 1 (PRODH1), which generates Glu by proline degradation under glucose- or Gln-limited conditions, further contributing to the energy metabolism and antioxidant capacity of the tumor cells (40). PDAC appear to have low or deficient expression of aspartate synthetase (Asn synthetase). As a result, they become dependent on exogenous aspartate supply, while plasma aspartate depletion using erythrocyte-encapsulated L-aspartase (ERY-ASP) becomes a potential therapeutic strategy. Metabolic levels of branched-chain amino acids (e.g., leucine, isoleucine, and valine) are significantly elevated in PDAC (41). This phenomenon is mainly due to enhanced proteolysis and reduced utilization of branched-chain amino acids by tumors mediated by TP53 mutations. In arginine metabolism, PDAC cells promote tumor growth by catabolizing arginine into urea and ornithine through high expression of arginase 2 (Arginase 2) (42). Reprogramming of various amino acid metabolisms provides PDAC cells with the necessary balance of biomolecular synthesis and reduction, and also enhances tumor growth and adaptation through complex molecular mechanisms.

### 2.3 Lipid metabolism in PC cells

**Figure 1:**
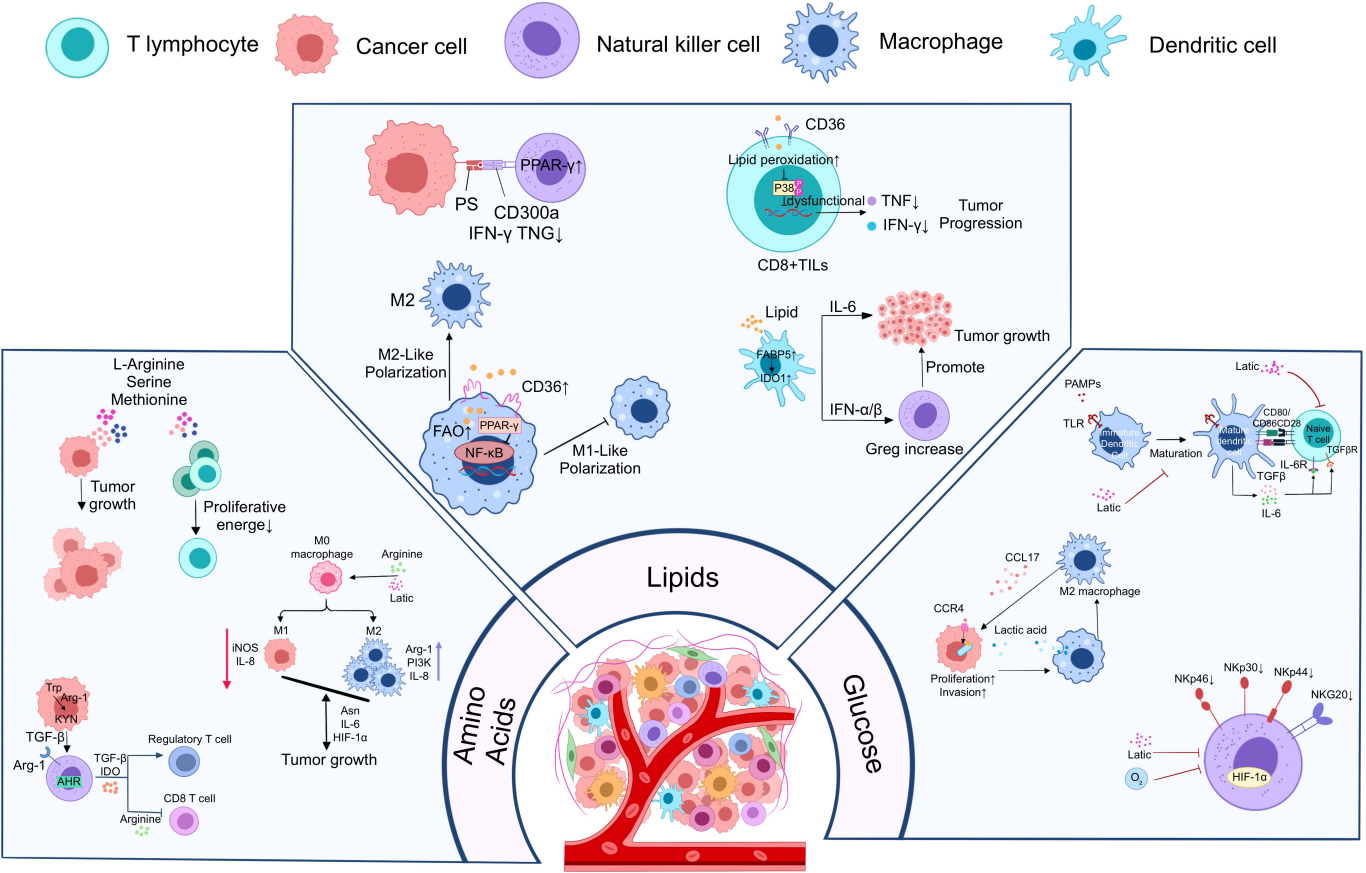
Metabolic reprogramming of PC cells to promote immune escape from tumors. PC cells promote tumor cell growth, proliferation, and metastasis through three major metabolic reprogramming modalities, including glucose metabolic reprogramming, amino acid metabolic reprogramming, and lipid metabolic reprogramming. Meanwhile, tumor cells enhanced their immune escape ability through the above metabolic reprogramming modalities and further inhibited the growth and function of anti-tumor immune cells.

In PDAC cells, reprogramming of lipid metabolism promotes rapid tumor cell proliferation, maintenance of membrane structural integrity, and adaptation to microenvironmental stress. Lipid metabolism mainly involves cholesterol metabolism and fatty acid metabolism. In terms of cholesterol metabolism, PDAC cells enhanced cholesterol biosynthesis and uptake by up-regulating 3-hydroxy-3-methylglutaryl coenzyme A reductase (HMGCR), ATP citrate cleavage enzyme (ACLY), fatty acid synthase (FASN), and sterol O-acyltransferase 1 (SOAT1), and thus enhanced cholesterol biosynthesis and uptake (43, 44). The negative feedback mechanism of cholesterol regulation is disrupted in PDAC cells, resulting in a significant upregulation of the expression of key regulators such as sterol regulatory element binding protein (SREBP). At the same time, the expression of liver X receptor (LXR) was significantly decreased, and the above series of changes promoted cholesterol synthesis and uptake (45). In addition, hypermethylation of the cholesterol 25-hydroxylase (CH25H) gene inhibited its expression, leading to cholesterol accumulation and promoting autophagic fusion process in lipid membranes (46). Overexpression of low-density lipoprotein receptor (LDLR) further supports tumor cell growth and progression by enhancing exogenous uptake of cholesterol (47). In contrast, preproteasome bacterial protease/kexin type 9 (PCSK9) regulates LDLR levels on the cell surface by mediating the internalization and degradation of LDLR, thereby affecting the efficiency of cholesterol uptake (48). In terms of fatty acid metabolism, PDAC cells significantly enhanced the dehydrogenation and synthesis of fatty acids. In PDAC, the expression of lipid metabolism-related enzymes such as acetyl coenzyme A carboxylase and fatty acid synthase, which catalyze the initial synthesis of fatty acids, was upregulated. In addition, KRAS mutations and hypoxic conditions contribute to increased uptake of exogenous monounsaturated fatty acids by PDAC cells. Their uptake of long-chain fatty acids is enhanced mainly by the fatty acid transporter protein CD36, which maintains cell membrane fluidity and signaling functions (49). Glutamate-oxaloacetate transaminase 2 (GOT2) acts as a fatty acid transporter protein in the nucleus, binds to PPARδ, and regulates the expression of genes related to lipid metabolism, such as cyclooxygenase 2 (COX2), to promote the transport and utilization of fatty acids (50). In addition, high expression of fatty acid esterase (ACAT-1) in PC cells promotes esterified storage of free cholesterol, while inhibition of ACAT-1 leads to accumulation of free cholesterol, which ultimately leads to severe endoplasmic reticulum stress and apoptosis of cancer cells (51). The use of statins (e.g., fluvastatin and simvastatin) as competitive inhibitors of HMGCR has been shown to inhibit cholesterol synthesis in PDAC cells and to enhance the effects of anticancer drugs by reducing PD-L1 expression (52). In addition, inhibitors of chelerythritol epoxygenase (SQLE), the second rate-limiting enzyme for cholesterol biosynthesis, such as terbinafine and NB-598 have shown potential to inhibit PDAC cell proliferation, induce cell cycle arrest, and activate ER stress-mediated apoptosis in preclinical studies (53). PDAC cells are programmed to satisfy their biosynthesis and basic energy needs through a fine and complex reprogramming of lipid metabolism, and also by regulating key metabolic enzymes and signaling pathways, ultimately enhancing the metabolic adaptation and survival of the tumor for metastasis. Here, we summarize in detail the metabolic reprogramming of PC cells (**Figure 1**).

## 3 Metabolic reprogramming in immunosuppressive microenvironments

**Figure 2:**
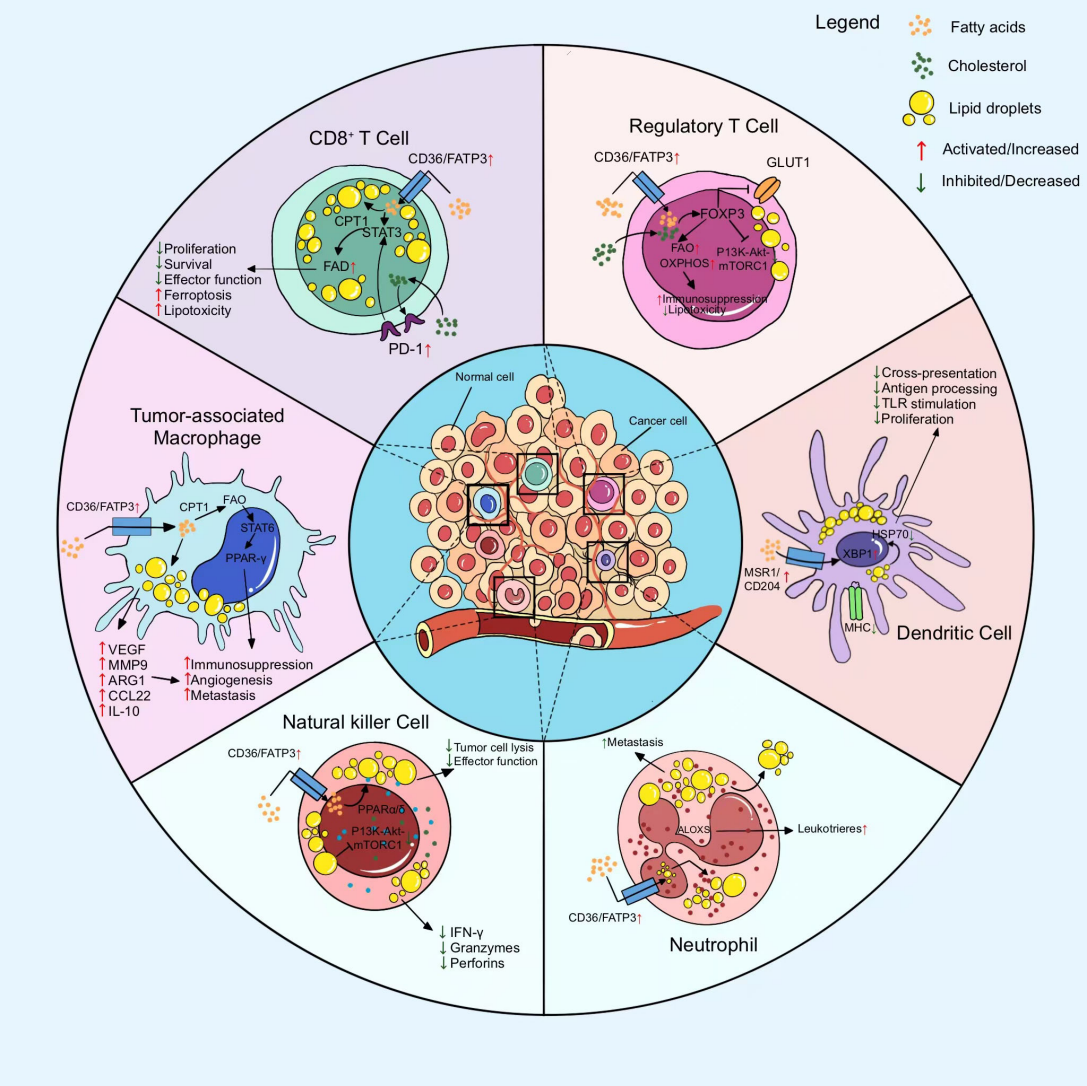
Metabolic reprogramming of major immune cells in the immunosuppressive microenvironment to suppress antitumor immune responses. Metabolic reprogramming of immune cells is mainly manifested in the inhibition of anti-tumor immune cell function and proliferation as well as the proliferation and growth of pro-tumor immune cells.

In TIME, anti-tumor immune cells such as CD8+T, DC, NK undergo aberrant metabolic reprogramming, resulting in restrictive inhibition of cell proliferation and function. In contrast, tumor-promoting immune cells such as M2-macrophages, Treg, CD4+T, etc. produce metabolic reprogramming that favors their proliferation and function. This unique metabolic reprogramming of immune cells ultimately induces the formation of TIME in PC. Metabolic reprogramming in the immunosuppressive microenvironment of PC (**Figure 2**).

### 3.1 T cell

In the TIME, a significant enhancement of glycolytic activity leads to a large accumulation of lactic acid in the TIME. The large accumulation of lactate suppresses anti-tumor immune responses by promoting the proliferation and function of Treg and inhibiting CD8+ T cell function. In T cells, first metabolic reprogramming enhances the immunosuppressive function of Treg. Tregs are capable of transporting exogenous lactate into the cell and ultimately converting it to phosphoenolpyruvate (PEP) by upregulating the lactate transporter protein MCT1 (54). During this process, the level of phosphoenolpyruvate in Tregs cells is significantly elevated, as well as the intracellular calcium ion concentration. It also promotes the nuclear translocation of nuclear factor-activated T cell 1 (NFAT1), which ultimately significantly upregulates the expression of PD-1 and enhances the immunosuppressive function in the TIME (55). In addition, the acidified environment resulting from the massive accumulation of lactate would also contribute to the metabolic shift of Tregs towards oxidative phosphorylation and increase nicotinamide adenine dinucleotide (NAD+) levels by activating the TGF-β signaling pathway (56). This series of processes ensures that Tregs can survive and proliferate under nutrient-deprived and acidic conditions, ultimately enhancing the immunosuppressive capacity of Tregs. In addition, Tregs maintain intracellular ROS homeostasis by elevating glutathione (GSH) levels, activating the NRF2 transcription factor, and enhancing their ability to survive in high-ROS environments through modulation of serine uptake (via the ASCT1 transporter) and maintenance of the expression of the key transcription factor FoxP3 (57). On the other hand, metabolic reprogramming inhibits the normal anti-tumor function of CD+8 T cells. Bone marrow-derived suppressor cells inhibit the mTOR signaling pathway by promoting L-arginine depletion, which in turn activates the GCN2-mediated amino acid starvation response and ultimately suppresses T cell growth and proliferation (58). Serine deficiency, on the other hand, affects the proliferative capacity of T cells, although not directly their activation and effector functions. In contrast, competitive depletion of methionine leads to demethylation of histone H3K79 in CD8+ T cells, decreases STAT5 expression, and impairs the immune response of T cells (59). In terms of lipid metabolism, activation of PD-1 in TME promoted fatty acid oxidation (FAO) in CD8+ T cells via the STAT3 signaling pathway, while inhibiting glycolysis and IFN-γ production (60). Leptin, secreted by adipocytes, is able to further enhance FAO and inhibit glycolysis in T cells through activation of the JAK2/STAT3 and mTOR signaling pathways, thereby impairing the antitumor activity of T cells. cD36 receptor-mediated uptake of oxidized low-density lipoproteins (OxLDL) leads to lipid peroxidation and activation of the p38 kinase pathway, which inhibits the T cell secretion of IFN-γ and TNF, thereby inhibiting their antitumor function (61). Lysophosphatidic acid (LPA) is also drastically inhibiting the normal function of T cells by disrupting the formation of the immune synapse (IS), interfering with the cytoskeletal organization of T cells and the localization of the inhibin type 1 receptor (IP3R1) (62). Linoleic acid (LA) uptake triggers mitochondrial oxidation and ROS generation in T cells, leading to cell death, to which CD4+ T cells are particularly sensitive, and promotes tumor cell growth (63). Metabolic reprogramming of T cells inhibits the proliferation and function of anti-tumor immune cells, such as CD+8T, while inducing the growth and proliferation of pro-tumor immune cells, such as CD+4T, which ultimately leads to the formation of an immunosuppressive microenvironment.

### 3.2 Macrophages

Macrophages are the most abundant cell type in the TIME with significant regulatory immune and metabolic functions. Tumor-associated macrophages (TAMs) polarize in response to a variety of stimuli in the TME and differentiate primarily into pro-inflammatory M1 and anti-inflammatory M2 types (64). M1-macrophages are induced by IFN-γ or LPS and have tumor suppressive functions, whereas M2-macrophages are stimulated by IL-4 and support tumor growth, angiogenesis and metastasis (65). The high lactate levels common in TME enter macrophages via monocarboxylate transporter proteins (MCT1-4), which activate the MCT-HIF1α signaling pathway and promote M2-type polarization (66). Lactate also further promotes M2-macrophages differentiation through activation of GPCRs and increased expression of cAMP and ICER, and these M2-macrophages secrete immunosuppressive molecules, such as IL-10 and TGF-β, to inhibit T cell activation (67, 68). In addition, lactate stimulates the mTOR signaling pathway, especially AKT (Ser473) and ERK, to promote M2 polarization. It also promotes PC cell growth and invasion by activating the mTORC1 pathway through CCL17 (69, 70). In a high glucose environment, TAMs elevated the migration potential of pancreatic ductal adenocarcinoma (PDAC) cells by secreting IL-8 and induced epithelial-mesenchymal transition (EMT). Succinic acid released by tumor cells enhances macrophage migration and promotes its polarization through activation of the succinic acid receptor (SUCNR1) while inducing EMT in cancer cells (71). The tryptophan metabolite, kynurenine (KYN), promotes M2 polarization through activation of the aryl hydrocarbon receptor (AhR) and inhibits M1 macrophage function through competitive uptake of tryptophan (72). In addition, tryptophan further inhibits antitumor function through CD36 receptor-mediated uptake of oxidized low-density lipoprotein (OxLDL), which leads to significant activation of lipid peroxidation and p38 kinase signaling pathways in macrophages, reducing cytokine secretion (73, 74). Metabolic reprogramming of M2 macrophages promotes their aberrant growth, proliferation, and function, whereas the growth and function of M1 macrophages are significantly consistently. Both results ultimately promote immune escape of tumor cells as well.

### 3.3 Dendritic cells

Dendritic cells (DCs) are inhibited from their anti-tumor functions through multiple metabolic reprogramming mechanisms. Efficient tumor cell glycolysis leads to lactate accumulation, which lowers the pH of the TME, interferes with the antigenic expression and presentation capacity of DCs, and contributes to the up-regulation of DCs surface molecules, such as CD86, and CD14 expression, which drive the shift to a macrophage-like phenotype (75, 76). In addition, immunosuppressive cytokines (e.g., IL-6 and IL-10) secreted by tumor cells impeded the maturation of DCs and reduced IL-12 production, further impairing antigen presentation. Lactic acid inhibits T-cell activation by activating the GPR81 receptor on the surface of DCs, altering intracellular signaling pathways, and decreasing the induction of type I interferon (77). Meanwhile, IDO enzyme-catalyzed tryptophan metabolism in TME increased kynurenine (Kynurenine) levels, activated the aryl hydrocarbon receptor (AHR), and promoted the differentiation of Tregs to enhance the immunosuppressive milieu (78, 79). TGF-β induced the expression of arginase 1 (ARG1) by DCs in TME, depleting arginines required for T cell activation and further inhibiting T cell function (80). In addition, it was found that: high-fat diet and lipid accumulation increased lipid uptake by DCs via macrophage scavenger receptor (MSR1), leading to defects in antigen processing and presentation. Oxidized lipids activate XBP1-mediated endoplasmic reticulum stress response, leading to lipid overload and significantly inhibiting DCs-mediated T cell activation by binding to heat shock protein 70 (Hsp70) and impeding the transport of peptide-MHC II complexes (81, 82). Metabolic reprogramming in TIME significantly inhibited the anti-tumor function of DCs through various mechanisms including lactate accumulation, altered amino acid metabolism and lipid overloading function and promoted immune escape from tumors. DCs play a critical role in antigen presentation and neoantigen generation. However, aberrant metabolic reprogramming inhibits the antigen-presenting function of DCs, leading to the failure of tumor cells to be recognized by anti-tumor immune cells, which in turn significantly enhances the immune escape of tumor cells.

### 3.4 NK cells

NK cells as an important component of the anti-tumor immune response, exert anti-tumor immune functions through the secretion of cytokines such as IFNγ and TNFα. NK cells kill tumor cells through the release of perforin and granzymes, or through death receptor-ligand interactions. However, metabolic changes in TME significantly affect NK cell function (83). High-fat diet and obesity showed a negative correlation with NK cell proliferation and their cytolytic activity against cancer cells in both human and mouse models, suggesting that lipid accumulation inhibits the effector function of NK cells. Prostaglandin E (Prostaglandin E) inhibits cytokine production and cytotoxicity of NK cells, and by inhibiting the prostaglandin E2 receptor EP4, the effector function of NK cells can be restored and tumor metastasis inhibited (84, 85). Exposure to lipid mixtures leads to impaired NK cell function. Overexpression of PPARα and PPARβ/δ promoted the expression of lipid transporter protein CD36 and low-density lipoprotein receptor, which increased the uptake and accumulation of lipids by NK cells, leading to a decrease in the secretion of effector cytokines by NK cells and a decrease in the ability of tumor cells to lyse (86). In contrast, the fatty acyl coenzyme A carboxylase 1 (CPT1) inhibitor etomoxir was able to restore cytotoxicity in NK cells (87). In addition, the SREBP family of transcription factors act as master regulators of lipid homeostasis, among which IL-2 and IL-12 play a key role in metabolic reprogramming of NK cells (88). They enhance IFNγ production by promoting glycolysis and activation of the citrate-malate shuttle system. In terms of amino acid metabolism, NK cells, similar to T cells, depend on arginine for rapid growth and proliferation (89). Arginine deficiency impairs NK cell cytotoxicity by inhibiting downstream signaling and reducing IFNγ expression. Although NK cells are less sensitive to arginine deficiency than T cells, arginine starvation still suppresses their IFNγ expression through post-transcriptional mechanisms. In addition, damage-associated molecular patterns from mitochondria (MitoDAMPs) deplete arginine in the TME, further impairing NK cell function and decreasing the expression of its activating receptor NKG2D (90, 91). However, exogenous arginine or arginase inhibitors can reverse the inhibitory effect of MitoDAMPs. Metabolic reprogramming of NK cells significantly inhibits their normal anti-tumor function, resulting in the inability to secrete cytokines such as IFNγ as well as IL-2 normally. This further has induced the formation of TIME.

## 4 Immunotherapy and metabolic reprogramming

Immunotherapy has become the newest therapeutic option, but its overall response rate is still less than 30%, and it faces problems such as large individual patient differences, drug resistance, and immune-related adverse effects. For patients with low response to immunotherapy, kynurenine levels can be reduced by targeting IDO/TDO inhibitors, which in turn inhibits Treg differentiation and reverses CD8+ T cell depletion in patients. Secondly, the use of ARG inhibitors can also restore the mitochondrial oxidative phosphorylation capacity of T cells and enhance the anti-tumor function of T cells. Further studies found that blocking the adenosine-CD73/A2AR axis restored the antigen-presenting function of DCs, which in turn greatly facilitated the ability of T cells to recognize tumors. Decreasing the acidity of the tumor microenvironment with lactate dehydrogenase inhibitors prior to the treatment of ICIs resulted in a 2.3-fold increase in T-cell infiltration in patients using PD-1 monoclonal antibody. Immunotherapy is rapidly developing as an emerging strategy, and several immunotherapeutic approaches have entered clinical trials. However, most clinical trials have been disappointing, showing limited efficacy. Recent studies have shown that the efficacy of T cell-mediated immunotherapy can be optimized by modulating cellular metabolism. Inhibition of the glycolytic enzyme PFKFB3 synergized with PD-1 blockers to enhance anti-tumor effects in mouse models of melanoma and colorectal cancer (92). In addition, inhibition of GLUT1 enhanced the therapeutic response to immune checkpoint inhibitors (ICIs) targeting PD-1 in a preclinical model of PC. Although GLUT3 overexpression partially compensated for GLUT1 inhibition, suggesting that dual GLUT1/GLUT3 inhibition may be the superior strategy (93). Inhibiting tryptophan-catabolizing enzymes IDO and TDO reduces kynurenine production, alleviates immunosuppression, and demonstrates synergistic potential with immune checkpoint inhibitors in clinical trials. A phase I/II clinical trial of the IDO inhibitor indoximod in combination with chemotherapy in patients with metastatic PC (NCT02077881) has been completed (94, 95). In terms of nucleotide metabolism, drugs that inhibit CD39, CD73, or adenosine receptors show potential for restoring anticancer immunity and improving disease control (96). The CD73 inhibitor AB680, in combination with a PD-L1 blocker, potentiated the efficacy of an immune checkpoint inhibitor in a mouse model of PC. In addition, adenosine A2a receptor (ADORA2A) antagonists synergize with a variety of immunotherapeutic strategies in a variety of preclinical tumor models, including CAR T-cell therapies in a leukemia model and PD-1 blockers in breast cancer and melanoma models (97). In lipid metabolism, inhibition of fatty acid oxidation (e.g., with the CPT1 inhibitor etomoxir) was shown to synergize with CD47-blocking antibodies and radiotherapy to enhance anti-tumor effects in a mouse glioblastoma (GBM) model (98). In addition, fatty acid synthase (FASN) inhibitors such as cerulenin and denifanstat restored the activation of dendritic cells (DCs) and tumor infiltration of effector T-cells in preclinical models and showed good tolerability and partial efficacy in clinical trials (e.g., NCT02980029, NCT03179904 et al.) (99). CD36 blockers have shown potential to synergize with immunotherapy in preclinical models of PC and melanoma, but their clinical development is in the early stages and only a few agents (e.g., VT1021) are under evaluation (49). NCT04471415 is a recent clinical trial of Gln as a therapeutic target and in combination with immunotherapy. In it, DRP-104 demonstrated significant therapeutic efficacy in combination with ICIs. The comprehensive treatment regimen of relevant drugs against metabolic targets in combination with immunotherapy has made great progress. Unfortunately, current combined metabolic-immunotherapies may trigger novel toxicities of therapy. It has been clinically observed that the use of immunotherapy in combination with IDO inhibitors induces neurotoxicity via the tryptophan-5HT axis in about 20% of patients and may exacerbate immune-associated myocarditis. With the use of PD-1 inhibitors in combination with adenosine receptor antagonists, IL-6 levels are significantly increased by a factor of 3, and the risk of cytokine storm production in patients is greatly increased. Here, we summarize clinical trials of targeted metabolic pathways combined with immunotherapy in PC (**Table 1**).

**Table 1:** Clinical trials of targeted metabolic pathways combined with immunotherapy in pancreatic cancer.

NCT Number	Study Title	Study Status	Conditions	Interventions	Phases
NCT06119217	Phase 2 Study of TTX-030 and Chemotherapy With or Without Budigalimab for 1L mPDAC Patients	ACTIVE_NOT_RECRUITING	Pancreatic Cancer	COMBINATION_PRODUCT: TTX-030, nab-paclitaxel and gemcitabine|COMBINATION_PRODUCT: TTX-030, budigalimab, nab-paclitaxel and gemcitabine|COMBINATION_PRODUCT: Nab-Paclitaxel and gemcitabine	PHASE2
NCT05692596	The Pancreas Interception Center (PIC) for Early Detection, Prevention, and Novel Therapeutics	RECRUITING	Pancreatic Ductal Adenocarcinoma|Pancreatic Cyst|Chronic Pancreatitis|Fatty Pancreas|Genetic Pancreatic Cancer|Genetic Pancreatitis|BRCA Mutation|Lynch Syndrome|FAP|Familial Atypical Multiple Mole-Melanoma|PALB2 Gene Mutation|Peutz-Jeghers Syndrome|Ataxia Telangiectasia	OTHER: Data collection	NA
NCT05617508	N-DOSE AD: A Dose Optimization Trial of Nicotinamide Riboside in Alzheimer’s Disease	RECRUITING	Alzheimer Disease|Dementia	OTHER: Placebo|DIETARY_SUPPLEMENT: Nicotinamide Riboside supplementation 1000mg daily in total|DIETARY_SUPPLEMENT: Nicotinamide Riboside dose escalation (up to 3000 mg daily in total)	PHASE2
NCT05562297	Neoadjuvant/Adjuvant Sintilimab, Nab-paclitaxel, and Gemcitabine for Resectable/Borderline Resectable Pancreatic Cancer	NOT_YET_RECRUITING	Pancreatic Cancer, Stage IB|Pancreatic Cancer, Stage IIA|Pancreatic Cancer, Stage IIB|Pancreatic Cancer Stage III	DRUG: sintilimab|DRUG: nab-paclitaxel|DRUG: gemcitabine	PHASE2
NCT04707365	Microenvironment and Immunity of Digestive Cancers - East Paris Multicentric Cohort	NOT_YET_RECRUITING	Colorectal Cancer|Pancreas Tumor|Biliary Tract Tumor|Immune System and Related Disorders	BIOLOGICAL: Tumor samples	NA
NCT04336098	Study of SRF617 in Patients With Advanced Solid Tumors	COMPLETED	Advanced Solid Tumor	DRUG: SRF617|DRUG: Gemcitabine|DRUG: Albumin-Bound Paclitaxel|DRUG: Pembrolizumab	PHASE1
NCT04306900	TTX-030 in Combination With Immunotherapy and/or Chemotherapy in Subjects With Advanced Cancers	COMPLETED	Solid Tumor, Adult	COMBINATION_PRODUCT: TTX-030, budigalimab and mFOLFOX6|COMBINATION_PRODUCT: TTX-030, budigalimab and docetaxel|COMBINATION_PRODUCT: TTX-030 and mFOLFOX6|COMBINATION_PRODUCT: TTX-030 and budigalimab|COMBINATION_PRODUCT: TTX-030, budigalimab, nab-paclitaxel and gemcitabine|COMBINATION_PRODUCT: TTX-030 and pembrolizumab|COMBINATION_PRODUCT: TTX-030, nab-paclitaxel and gemcitabine|COMBINATION_PRODUCT: Budigalimab and mFOLFOX6	PHASE1
NCT03884556	TTX-030 Single Agent and in Combination With Immunotherapy or Chemotherapy for Patients With Advanced Cancers	COMPLETED	Solid Tumor|Lymphoma	DRUG: TTX-030|DRUG: Pembrolizumab|DRUG: Gemcitabine|DRUG: nab paclitaxel	PHASE1

## 5 Limitations and future directions

### 5.1 Limitations and challenges

Combined immunotherapy and metabolic reprogramming strategies are still facing great challenges. First, metabolic reprogramming in different tumors and immune cells has great heterogeneity and complexity, and the screening of mechanistic targets is very difficult. Second, the issue of synergistic toxicity between immunotherapy and metabolic drug therapy remains a great challenge. Although the efficacy is increased by the combination, the incidence of adverse effects such as hypertension and proteinuria is gradually increasing. In addition, there is a lack of individualized treatment protocols, which prevents patients from receiving precise treatment due to the lack of accurate multi-omics data. Finally, the huge difference between animal models and humans in clinical translation and the lack of long-term efficacy validation still need to be focused on.

### 5.2 Future directions

Clinical regimens using metabolic reprogramming as a drug target and combining it with immunotherapy have a broad prospect in the field of tumor therapy. First, by targeting metabolic reprogramming, the efficacy of immunotherapy can be improved by regulating energy metabolism and the normal function of immune cells in the tumor immune microenvironment. We focus on exploring the mechanisms of mitochondrial oxidative phosphorylation reactivation and glutamine metabolism regulation to reverse T cell depletion. In PC, cholesterol metabolic reprogramming by targeting NPC1L1 could reverse the functional inhibition of CD8+ T cells, and the combination of ezetimibe with anti-PD-1 immunotherapy could significantly enhance the therapeutic efficacy. Second, the future direction of combined immunotherapy and metabolic reprogramming focuses on the application of multi-omics technologies. With the continuous development of sequencing technology, multi-omics technology can deeply analyze the interplay mechanism between metabolism and immunity in the tumor microenvironment and provide theoretical support for precise treatment. Meanwhile, focusing on the problem of drug resistance, we will develop new nanoscale drug carriers to realize the targeted co-delivery of metabolic drugs and immunotherapeutic drugs. We will establish organ chips and organ-like models to simulate the tumor-immunity-metabolism tripartite interaction network. Instead of being limited to only two-dimensional cell types.

## 6 Conclusion

Metabolic reprogramming interacts through multiple mechanisms in the tumor suppression microenvironment and collectively promotes immunosuppression of tumor cells. Targeting glucose metabolism, amino acid metabolism and lipid metabolism, an increasing number of studies have found that all types of cells in the tumor microenvironment undergo metabolic alterations according to different metabolic demands. Although some metabolic targets have demonstrated good synergistic effects in preclinical studies, the therapeutic effects are not satisfactory in clinical applications. The complexity of metabolic pathways and the dependence of healthy cells on metabolic inhibition make the combination with immunotherapy a major challenge. In the future, the focus should be on the development of highly targeted and selective metabolic modulation strategies in combination with existing immunotherapeutic approaches to significantly enhance the efficacy of cancer treatment. It is believed that targeting metabolic modulation targets can provide new research directions and potential therapeutic targets to overcome immunosuppression in TME and enhance the efficacy of immunotherapy in the near future.

## Glossary

PC: pancreatic cancer
TME: tumor microenvironment
TIME: tumor immune microenvironment
OXPHOS: oxidative phosphorylation
IDO: indoleamine 2,3-dioxygenase
TDO: tryptophan 2,3-dioxygenase
Kyn: kynurenine
PD-1: programmed cell death protein 1
CTLA-4: cytotoxic T-lymphocyte-associated protein 4
GLUT1: glucose transporter type 1
HK1/2: hexokinase 1/2
LDHA: lactate dehydrogenase A
PDHK1: pyruvate dehydrogenase kinase 1
PDH: pyruvate dehydrogenase
PPP: pentose phosphate pathway
HBP: hexosamine biosynthesis pathway
GFPT1: glutamine-fructose-6-phosphate amidotransferase-1
Nampt: nicotinamide phosphoribosyltransferas
NAD: nicotinamide adenine dinucleotide
PGK1: phosphoglycerate kinase 1
TIGAR: TP53-induced glycolysis and apoptosis regulator
GAPDH: glyceraldehyde-3-phosphate dehydrogenase
HIF-1: hypoxia-inducible factor-1
PKM2: muscle isomerase type 2 pyruvate kinase
FOXM1: Forkhead box protein M1
NUSAP1: nucleolus and spindle-associated protein 1
H3K18la: histone H3 lysine 18 lactylation
Gln: glutamine
Glu: glutamate
GLS1: glutaminase 1
GOT1: glutamate oxaloacetate transaminase 1
OAA: oxaloacetic acid
α-KG: α-ketoglutarate
Asp: aspartic acid (Asp)
MDH1: malate dehydrogenase 1
ROS: reactive oxygen species
CARM1: coactivator-associated arginine methyltransferase 1
GLS2: glutaminase 2
PRODH1: proline oxidase 1
ARG2: arginase 2
HMGCR: 3-hydroxy-3-methylglutaryl coenzyme A reductase
ACLY: ATP citrate lyase
FASN: fatty acid synthase
SOAT1: sterol O-acyltransferase 1
SREBP: sterol regulatory element binding protein
LXR: liver X receptor
CH25H: cholesterol 25-hydroxylase
LDLR: low-density lipoprotein receptor
PCSK9: proprotein convertase subtilisin/kexin type 9
ACC: acetyl-CoA carboxylase
CD36: fatty acid transporter protein
COX2: cyclooxygenase 2
ACAT-1: acyl-CoA cholesterol acyltransferase-1
SQLE: squalene epoxidase
MCT1: monocarboxylate transporter 1
PEP: phosphoenolpyruvate
NFAT1: nuclear factor of activated T cells 1
NRF2: nuclear factor erythroid 2-related factor 2
ASCT1: alanine-serine-cysteine transporter 1
FoxP3: forkhead box P3
FAO: fatty acid oxidation
STAT3: signal transducer and activator of transcription 3
JAK2: Janus kinase 2
OxLDL: oxidized low-density lipoprotein
LPA: lysophosphatidic acid
IP3R1: inositol 1,4,5-trisphosphate receptor 1
LA: linoleic acid
TAMs: tumor-associated macrophages
LPS: lipopolysaccharide
GPCR: G protein-coupled receptor
ICER: inducible cAMP early repressor
mTOR: mechanistic target of rapamycin
AKT: protein kinase B
ERK: extracellular signal-regulated kinase
CCL17: C-C motif chemokine ligand 17
EMT: epithelial-mesenchymal transition
SUCNR1: succinate receptor
AhR: aryl hydrocarbon receptor
DCs: dendritic cells
IL: interleukin
GPR81: G protein-coupled receptor 81
ARG1: arginase 1
MSR1: macrophage scavenger receptor 1
XBP1: X-box binding protein 1
HSP70: heat shock protein 70
NK: natural killer
IFNγ: interferon gamma
TNFα: tumor necrosis factor alpha
PGE: prostaglandin E
PPARα: peroxisome proliferator-activated receptor alpha
PPARβ/δ: peroxisome proliferator-activated receptor beta/delta
CPT1: carnitine palmitoyltransferase 1
NKG2D: natural killer group 2D
DAMPs: damage-associated molecular patterns
MitoDAMPs: mitochondrial DAMPs
PFKFB3: phosphofructokinase-2/fructose-2,6-bisphosphatase 3
CD73: ecto-5’-nucleotidase
ADORA2A: adenosine A2a receptor
CAR: chimeric antigen receptor
SCAP: SREBP cleavage-activating protein.
