# Correction: Crosstalk between the tumor immune microenvironment and metabolic reprogramming in pancreatic cancer: new frontiers in immunotherapy

**DOI:** 10.3389/fimmu.2025.1643932

**Published:** 2025-07-25

**Authors:** Taijin Shi, Xiaoyan Cui, Junlin Wang, Guangqia Liu, Jiayin Meng, Yingjie Zhang

**Affiliations:** ^1^ College of Medicine, Shandong University of Traditional Chinese Medicine, Jinan, China; ^2^ Pharmacy Department, Jinan Huaiyin People’s Hospital, Jinan, China; ^3^ Department of Pharmacy, Shandong Second Provincial General Hospital, Jinan, China; ^4^ Department of Pharmacy, Jinan Licheng District Liubu Town Health Centre, Jinan, China; ^5^ Department of Pharmacy, Jinan Second People’s Hospital, Jinan, China

**Keywords:** PC, time, metabolic reprogramming, mechanisms, immunotherapy

There was an error in affiliation 3. Instead of “Department of Pharmacy, Shandong University Second People’s Hospital, Jinan, China”, it should be “Department of Pharmacy, Shandong Second Provincial General Hospital, Jinan, China”.

Author “Taijin Shi” was erroneously spelled as “Jintai Shi”.

The Author Contributions Statement has been corrected to read:

“TS: Writing – original draft, Writing – review & editing. XC: Writing – original draft, Writing – review & editing. JW: Supervision, Writing – review & editing. GL: Methodology, Writing – original draft. JM: Validation, Writing – original draft. YZ: Supervision, Writing – original draft, Writing – review & editing.”

The original version of this article has been updated.

